# Probing Protein-Protein Interactions Using Asymmetric Labeling and Carbonyl-Carbon Selective Heteronuclear NMR Spectroscopy

**DOI:** 10.3390/molecules23081937

**Published:** 2018-08-03

**Authors:** Erik K. Larsen, Cristina Olivieri, Caitlin Walker, Manu V.S., Jiali Gao, David A. Bernlohr, Marco Tonelli, John L. Markley, Gianluigi Veglia

**Affiliations:** 1 Department of Chemistry, University of Minnesota, Minneapolis, MN 55455, USA; larse585@umn.edu (E.K.L.); mvelipar@umn.edu (M.V.S.); jgao@umn.edu (J.G.); 2 Department of Biochemistry, Molecular Biology, and Biophysics, University of Minnesota, Minneapolis, MN 55455, USA; colivier@umn.edu (C.O.); walk0512@umn.edu (C.W.); bernl001@umn.edu (D.A.B.); 3 National Magnetic Resonance Facility at Madison, Madison, WI 53706, USA; tonelli@nmrfam.wisc.edu; 4 Department of Biochemistry, University of Wisconsin-Madison, Madison, WI 53706, USA; markley@biochem.wisc.edu

**Keywords:** protein-protein interactions (PPI), nuclear magnetic resonance (NMR), Carbonyl Carbon Label Selective (CCLS), dual carbon label selective (DCLS), residual dipolar coupling (RDC), paramagnetic relaxation enhancement (PRE)

## Abstract

Protein-protein interactions (PPIs) regulate a plethora of cellular processes and NMR spectroscopy has been a leading technique for characterizing them at the atomic resolution. Technically, however, PPIs characterization has been challenging due to multiple samples required to characterize the hot spots at the protein interface. In this paper, we review our recently developed methods that greatly simplify PPI studies, which minimize the number of samples required to fully characterize residues involved in the protein-protein binding interface. This original strategy combines asymmetric labeling of two binding partners and the carbonyl-carbon label selective (CCLS) pulse sequence element implemented into the heteronuclear single quantum correlation (^1^H-^15^N HSQC) spectra. The CCLS scheme removes signals of the J-coupled ^15^N–^13^C resonances and records simultaneously two individual amide fingerprints for each binding partner. We show the application to the measurements of chemical shift correlations, residual dipolar couplings (RDCs), and paramagnetic relaxation enhancements (PRE). These experiments open an avenue for further modifications of existing experiments facilitating the NMR analysis of PPIs.

## 1. Introduction

Biological processes rely primarily on protein-protein interactions (PPIs) to mediate a cellular function [1]. Historically biochemical techniques (co-immunoprecipitation, yeast-two hybrid, pull-down assays, etc.) measuring parameters intrinsic to the whole complex have been used to characterize these PPIs [2,3]. Recently, advances in nuclear magnetic resonance (NMR) spectroscopy have provided the means to characterize PPIs at an atomic resolution, which offers fine details of individual macromolecules participating within the complex [4,5,6]. In addition to allowing the characterization of these complexes at an atomic resolution, NMR is well-suited for studying dynamic, transient (~100 μM K_D_), and low-populated states of complexes [7,8,9].

The mapping of PPIs using several observables such as chemical shift perturbation (CSP), residual dipolar couplings (RDC), intra-molecular and inter-molecular as well as solvent paramagnetic relaxation enhancement (PRE) [10,11,12], cross-saturation (CS), and nuclear Overhauser effects (NOEs) has been well-established [5]. These methods, nevertheless, fall short when studying large complexes due to the inherent attenuation of transverse relaxation times (T_2_), which results in a reduction of both signal intensity and resolution. Despite methods such as TROSY [13], deuteration [14,15], and selective labeling [16,17] addressing these concerns, multiple samples are still required to distinguish one species from another. Recently, several new NMR experiments based on simultaneous, interleaved detection of up to three NMR active species with distinct isotopic labeling have provided the opportunity to map the effect of PPIs on individual components within a macromolecular complex. While there are outstanding reviews on protein-protein interactions [18,19,20,21,22,23,24], we focus on our recently developed method that exploits the spin-echo filtering-based experiments with strategic protein labeling schemes to characterize protein-protein complexes.

## 2. Results

### 2.1. Mapping Two Binding Partners Fingerprints Simultaneously

**Figure 1:**
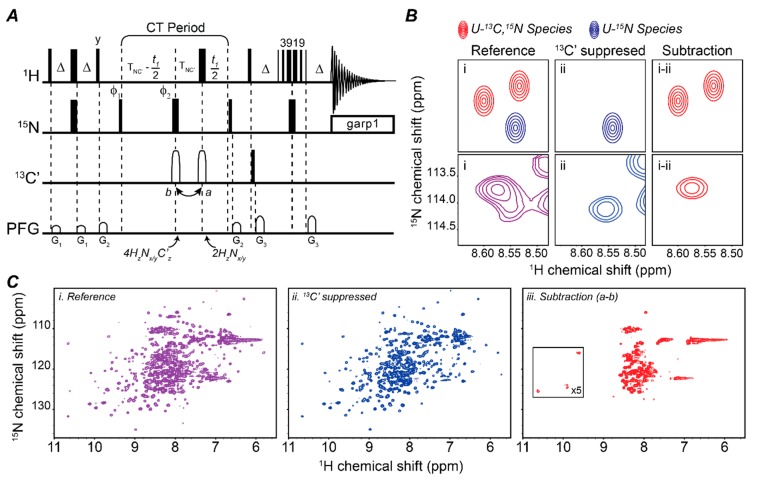
CCLS pulse sequence. (**A**) Schematic of the CCLS-HSQC pulse sequence. It can be assumed, unless otherwise indicated, that all rectangular pulses are applied along the *x*-axis. 90° and 180° flip angles are represented by narrow bars and wide bars, respectively. The carrier frequency for ^1^H is set on resonance with water at 4.77 ppm. The carrier frequency for ^15^N is set in the center of the amide region at 121.8 ppm. The ^13^C offset is set to 174.8 for the C′ region. The reference spectrum is recorded with the shaped pulse for ^13^C′ (open rectangle) at the position while the suppression spectrum is recorded with this pulse in position *b*. A 3-9-19 Watergate pulse scheme is used in the reverse INEPT transfer. GARP1 decoupling with a field strength of 1 kHz is used during the acquisition of ^15^N. Delay durations: Δ = 2.4 ms, δ = 0.11 ms, *T*_NC′_ = 16.5 ms. Phase cycling: φ_1_ = x, −x, φ_2_ = x, x, −x, −x, φ_rec_ = x, −x. A second FID is acquired for each increment by changing the φ_1_ phase to y, −y in order to accomplish States quadrature detection for the ^15^N indirect dimension. The φ_1_ and φ_rec_ phases are also incremented by 180° every other ^15^N increment for States-TPPI acquisition. The gradients use the Wurst shaped *z*-axis gradients of 1 ms. Gradient strengths (G/cm): G1: 5, G2: 7, G3: 17. The CCLS-HSQC pulse sequence is based on the fast HSQC experiment [44] to preserve water magnetization. (**B**) Example spectra representing the reference spectrum, the suppression spectrum, and the resulting subtraction spectrum followed by insets from the PKA-C/PKIα complex displaying the separation of resonances from each species. The blue and red species are present in the reference CCLS-HSQC while the suppression spectrum contains only blue species. Subtraction of the suppression from the reference spectrum results in a third spectrum containing only the red species. (**C**) CCLS-HSQC experiment on the 50 kDa PKA-C/PKIα complex. The reference spectrum (left, purple) displays resonances from both U-^15^N labeled PKA-C as well as U-^15^N, ^13^C labeled PKIα (S/N = 40). The suppression spectrum (middle, blue) suppresses a signal from the ^13^C′ labeled PKIα, which shows only peaks from ^12^C′ labeled PKA-C (S/N = 50). Upon subtraction of the suppression spectrum from the reference spectrum, a sub-spectrum is obtained containing only peaks from the PKIα (right, red) (S/N = 15). (All figures were cited with permission of Springer Nature).

Traditional approaches to map PPIs at an atomic level involve repeat experiments with reverse labeling patterns such that the interaction is probed from both binding partners. Prior to the introduction of the spin-echo filtering experiment by Bax et al. [25], three-bond homonuclear ^1^H-^1^H *J* couplings had been used to derive backbone and side-chain conformational restraints [26,27,28,29,30,31]. This new experiment relied on measuring the magnetization loss due to unresolved *J* coupling and utilized an interleaved detection method where two spectra are recorded simultaneously but differ by 180° pulse positions on the ^15^N channel. The spin-echo filtered experiment introduced by Bax is the building block for the Carbonyl Carbon Label Selective (CCLS) ^1^H-^15^N HSQC pulse sequence [32], which requires specific isotopic labeling to simultaneously map the chemical shift perturbations from two binding partners. The CCLS ^1^H-^15^N HSQC pulse sequence utilizes spin-echo filtering with a short magnetization transfer period between ^15^N and ^13^C′ in order to detect ^1^H-^15^N correlations adjacent to the NMR inactive (^12^C′) carbonyl groups (Figure 1A). Building on the constant time (CT) HSQC, the CCLS ^1^H-^15^N HSQC necessitates the acquisition of two spectra, a reference spectrum, and a suppression spectrum in an interleaved manner. The reference spectrum is acquired using the pulse sequence reported in Figure 1A with the 180° pulse on ^13^C′ during the ^15^N CT evolution period applied at position a as proposed by Vuister et al. [25], which allows for the removal of ^13^C′-^15^N coupling. The suppression spectrum is acquired with the 180° ^13^C′ pulse at position b, leaving ^13^C-^15^N *J* coupling active, and converting the transverse in-phase magnetization of ^15^N spins linked to ^13^C′ to antiphase magnetization. This antiphase magnetization contains components in both the *x*-direction and *y*-direction. The π ^1^H and ^13^C pulses applied at the end of the ^15^N evolution convert the *y*-component, 4H_z_N_y_C′_z_, to an unobservable multiple quantum coherence, 4H_y_N_z_C′_y_, while the *x*-component, 4H_y_N_z_C’_y_, is de-phased by the G2 gradient (Figure 1A). As a result, signals from ^1^H-^15^N groups coupled to ^13^C′ are suppressed while signals from ^1^H-^15^N groups coupled to ^12^C′ are unaffected. The suppression spectrum can then be subtracted from the reference spectrum, which leaves the U-^15^N, ^13^C species observable (Figure 1B).

We tested the sensitivity of the CCLS method by comparing a reference CCLS-HSQC spectrum and a conventional HNCO spectrum of the 20-kDa protein U-^13^C, ^15^N-Ubiquitin at 10 °C, 20 °C, 30 °C, and 40 °C corresponding to average *T*_2_ values of 27 ms, 33 ms, 40 ms, and 47 ms, respectively [32]. The slower tumbling rates at lower temperatures lead to longer rotational correlation times (*τ_C_*) and faster relaxation results in broader linewidths. We found the reference CCLS-HSQC experiment was more sensitive compared to the HNCO experiment for lower temperatures, which demonstrates that the shorter time delay (*T*_NC_) allows for increased sensitivity for large proteins or protein-protein complexes. The sensitivity enhancement gained from optimal *T*_NC_ values compensates for the decrease in S/N observed upon subtraction of the suppression spectrum from the reference spectrum.

Furthermore, we applied this technique to resolve assignment ambiguities on the 41 kDa catalytic subunit of cAMP-dependent protein kinase A (PKA-C) [33,34]. PKA-C is the prototypical Ser/Thr kinase and, until relatively recently, had remained unexplored by NMR due to its size and presence of conformational exchange effects on the μs-ms timescale [35,36,37,38]. Advances in pulse sequence design and sample preparation have since made it possible to investigate this system using NMR [39,40,41,42]. We successfully implemented the CCLS-HSQC pulse sequence to assist in the assignment of multiple catalytically relevant residues of PKA-C. Furthermore, recent work from our group demonstrates the ability of this pulse sequence to simultaneously detect PKA-C in complex with an endogenous inhibitor known as the heat-stable protein kinase A inhibitor (PKIα) [34,43], which gives the possibility to detect the mutual effect of PKA-C and PKIα interaction (Figure 1C—unpublished data). Together, these applications underscore the ability of CCLS to simultaneously detect PPIs and emphasizes the performance of this pulse sequence with high molecular weight systems.

### 2.2. Fingerprinting Three Binding Partners Using One Sample 

**Figure 2:**
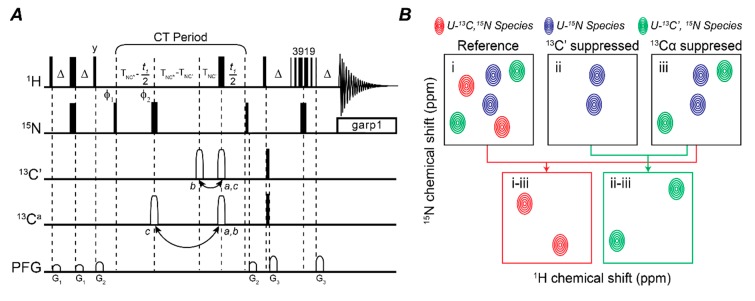
DCLS Pulsepulse sequence. (**A**) Schematic of the DCLS-HSQC pulse sequence. It can be assumed, unless otherwise indicated, that all pulses are applied along the *x*-axis. 90° and 180° flip angles are represented by narrow bars and wide bars, respectively. The reference spectrum is recorded with the shaped pulse for ^13^C (open rectangle) at position a while the ^13^C′ suppression spectrum is recorded with this pulse in position b and the ^13^C_α_ suppression spectrum is recorded with the ^13^C_α_ shaped pulse in position c. A 3-9-19 water-gate pulse scheme is used in the reverse INEPT transfer. GARP1 decoupling with a field strength of 1 kHz is used during the acquisition of ^15^N. The carrier frequency for ^1^H is set on the resonance with water at 4.7 ppm. The carrier frequency for ^15^N is set in the center of the amide region at 120 ppm and the ^13^C offset is set to 56 ppm. Selective ^13^C′ (^13^C_α_) sine shaped pulses are centered at 174 (56 ppm) and a null 113 ppm away. Delay durations: Δ = 2.4 ms, δ = 0.11 ms, *T*_NC′_ = 16.4 ms, and *T*_NC__α_ = 24.5 ms. Phase cycling: φ_1_ = x, −x, φ_2_ = x, x, −x, −x, φ_rec_ = x, −x. A second FID is acquired for each increment by changing the φ_1_ phase to y, −y in order to accomplish states quadrature detection for the ^15^N indirect dimension. The φ_1_ and φ_rec_ phases are also incremented by 180° every other ^15^N increment for States-TPPI acquisition. The gradients use the Wurst shaped *z*-axis gradients of 1 ms. Gradient strengths (G/cm): G1: 5, G2: 7, G3: 17. (**B**) Example spectra representing the reference spectrum, the two suppression spectra, and the resulting subtraction spectra. The red, green, and blue species are present in the reference of DCLS-HSQC (a) while the first suppression spectrum (b) contains resonances from the blue species and the second suppression spectrum (c) contains resonances from the blue and green species. Subtracting spectrum c from a results in only resonances from the red species and subtracting spectrum c from b yields only resonances from the green species. This linear subtraction scheme results in spectra with each component in the mixture isolated. (All figures were cited with permission of American Chemical Society).

Masterson et al. applied the CCLS pulse sequence element to deconvolute PPIs in a ternary mixture simultaneously [45]. The dual carbon label selective (DCLS) ^1^H-^15^N HSQC experiment requires three labeled binding partners with the first species U-^15^N labeled, the second ^15^N, ^13^C′ labeled, and the third U-^13^C, ^15^N labeled. The deconvolution of these spectra follows the same spin-echo filtering theory as CCLS with additional filtering of Cα coupled spins (Figure 2A). Cα suppression requires a longer *T*_NCα_ delay due to both inter-residue and intra-residue ^1^H-^13^Cα *J* coupling [46]. Increasing the *T*_NCα_ delay nullifies protein backbone conformation dependency of ^1^*J*_NCα_ and ^2^*J*_NCα_ since it completely suppresses the signal from ^1^*J*_NCα_ while inverting the residual signal intensities of ^2^*J*_NCα_.

This and the previously introduced pulse sequence rely on selective labeling of individual binding partners. Asymmetric selective labeling schemes to study PPIs in a multiple component sample are increasing in popularity [16,47,48,49] both for solution and solid-state NMR spectroscopy. For instance, Anglister and coworkers have demonstrated the application of asymmetric deuteration in combination with transferred nuclear Overhauser spectroscopy to study intermolecular nuclear Overhauser effects (NOEs) of large, fast exchanging protein complexes [50,51,52]. With respect to CCLS and DCLS, selective labeling of ^13^C′ can be accomplished in recombinant proteins using either ^15^N- and ^13^C′-labeled amino acids or 1-^13^C pyruvate and ^13^C-labeled NaHCO_3_ as the sole carbon sources [53,54,55,56,57]. Selective ^13^Cα labeling is achieved by using 2-^13^C glucose as the sole carbon source [54].

The DCLS experiment requires the acquisition of three interleaved experiments in parallel (Figure 2B). A reference data set is collected observing all three species simultaneously, which is followed by the first suppression data set where amide resonances adjacent to ^13^C′ are undetected. This is identical to the CCLS suppression spectrum. Lastly, a second suppression data set is collected where amide resonances coupled to ^13^Cα are not detected. Deconvolution of the spectra is obtained by a linear combination of the data set. Subtraction of the second suppression spectrum from the reference spectrum provides a sub-spectrum containing only resonances from the U-^13^C, ^15^N labeled species. The subtraction of the first suppression spectrum from the second suppression spectrum provides an additional sub-spectrum containing only resonances from the U-^15^N, ^13^C′ labeled species. In this manner, sub-spectra are obtained from a single sample for each individual component of the ternary mixture and all resonances can be resolved. As a proof of concept, Masterson et al. applied this labeling scheme and pulse sequence to three non-interacting proteins, which includes maltose binding protein (MBP), Kemptide, and ubiquitin. By applying DCLS, the authors obtained sub-spectra corresponding to each individual component of the ternary mixture displaying the potential of this approach to study protein-protein interactions with a single sample.

### 2.3. Measuring Residual Dipolar Coupling (RDC) of Complexes Using One Sample

**Figure 3:**
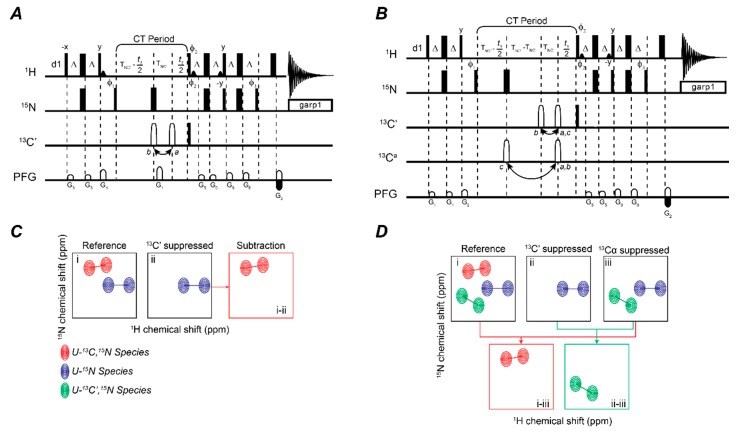
CCLS/DCLS RDC. (**A**,**B**) Schematic of the gradient-selected TROSY-based pulse sequence for binary (**A**) or ternary (**B**) mixtures of proteins. It can be assumed, unless otherwise indicated, that all pulses are applied along the *x*-axis. 90° and 180° flip angles are represented by narrow bars and wide bars respectfully. The reference spectrum is recorded with the shaped pulse for ^13^C (open rectangle) at position *a* while the ^13^C′ suppression spectrum is recorded with this pulse in position b and the ^13^C_α_ suppression spectrum is recorded with the ^13^C_α_ shaped pulse in position c. The carrier frequency for ^1^H is set on resonance with water at 4.77 ppm, the carrier frequency for ^15^N is set in the center of the amide region at 121.8 ppm, and the ^13^C offset is set to 56 ppm. Selective ^13^C′ (^13^C_α_) sine shaped pulses are centered at 174.8 ppm (56 ppm). Delay durations: Δ = 2.4 ms, δ_1_ = 1.5 s, *T*_NC′_ = 16.5 ms, and *T*_NC__α_ = 23.5 ms. Phase cycling: φ_1_ = x, −x, φ_2_ = −x, φ_3_ = −y, φ_rec_ = x, −x. Gradient strengths must be adjusted following the relationship G_2_ = G_1_·(γ_N_/γ_H_) where γ_N_ and γ_H_ are the gyromagnetic ratios of ^15^N and ^1^H, respectively. A second FID is collected for each increment by changing the φ_2_ and φ_3_ to x and y, respectively, and by inverting the sign of the G_2_ gradient in order to accomplish states quadrature detection for the ^15^N indirect dimension. The φ_1_ and φ_rec_ phases are also incremented by 180° with every other ^15^N increment for states-TPPI acquisition. The gradients use the Wurst shaped *z*-axis gradients of 1 ms. Gradient strengths (G/cm): G_3_: 3, G_4_: 13, G_5_: 4, G_6_: 5. To measure ^1^*J*_HN_ coupling and NH RDC (in aligned media), which is a second spectrum, featuring the anti-TROSY component is acquired by changing the φ_3_ phase to y. (**C**) Example spectra representing the reference spectrum, the suppression spectrum, and the resulting subtraction spectrum. The blue and red species are present in the reference CCLS-HSQC while the suppression spectrum contains only blue species. Subtraction of the suppression from the reference spectrum results in a third spectrum containing only the red species. (**D**) Example spectra representing the reference spectrum, the two suppression spectra, and the resulting subtraction spectra. The red, green, and blue species are present in the reference DCLS-HSQC (a) while the first suppression spectrum (b) contains resonances from the blue species and the second suppression spectrum (c) contains resonances from the blue and green species. Subtracting spectrum c from results in only resonances from the red species and subtracting spectrum c from b yields only resonances from the green species. This linear subtraction scheme results in spectra with each component in the mixture isolated. (All figures were cited with permission of American Chemical Society).

Residual dipolar coupling (RDC) allows orientation specific data to be derived through dipole-dipole interactions. The orientation restraints provided by RDC have proven useful in protein structure determination, nucleic acid structure, domain orientation, and more recently PPIs [58,59]. We implemented CCLS and DCLS to sensitivity-enhanced TROSY or anti-TROSY spin-state selection to record the simultaneous measurement of RDCs [58,60,61,62,63] for the relative orientations of multiple proteins within a single sample (Figure 3A,B). RDC measurements are susceptible to experimental conditional variations, which alter alignment tensors, making direct correlations of orientational constraints obtained from different samples more difficult. Our approach, together with specific isotopic labeling, eliminates the need for multiple samples and, therefore, removes errors associated with sample inconsistencies [58].

Similar to DCLS (Figure 3C), we applied this pulse sequence to a non-interacting mixture of U-^2^H, ^15^N MBP, ^15^N-Ser^5^, ^13^C′-Ala^4^ Kemptide, and, U-^13^C, ^15^N ubiquitin [64]. Following the same linear subtraction scheme reported for DCLS, we were able to measure RDCs for each individual component in a ternary mixture. Furthermore, these RDC values were in agreement with back calculated values determined from already solved crystal structures of MBP [65] and ubiquitin [66], which confirms that the backbone conformational space of these proteins along with their relative alignment tensors were sufficiently defined.

### 2.4. Measuring Long-Range Distances and Transient Complexes Using CCLS for Paramagnetic Relaxation Enhancements (PRE)

**Figure 4:**
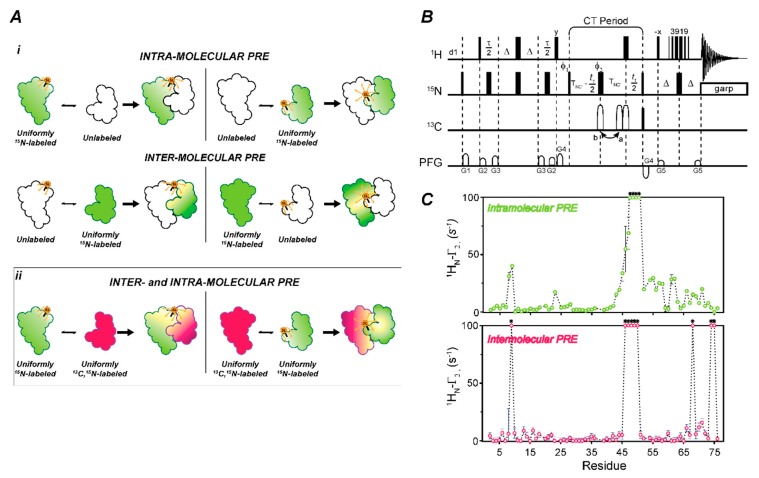
CCLS-PRE. (**A**) Schematic of the standard experiment for the detection of intra-molecular and inter-molecular PRE. In this case, four different samples are needed. The first sample for the intra-molecular PRE is prepared with an asymmetric labeling scheme using the first binding partner uniformly ^15^N labeled with a conjugated spin label (SL) and the second is NMR silent (unlabeled). A sample with a reversed labeling scheme is necessary to detect the intra-molecular PRE for the second binding partner (top panel **A**). For the inter-molecular PRE, two additional samples are required: one NMR silent with the conjugated SL and a second NMR active (e.g., ^15^N or ^13^C labeled) (lower panel **A**). Simultaneous detection of inter-molecular and intra-molecular PRE using ^1^H_N_-*Γ*_2_-CCLS experiment. One species is uniformly ^15^N labeled while the other is ^13^C and ^15^N labeled (b) allowing for the simultaneous detection of intra-molecular and inter-molecular PREs. As reported before, reverse positioning of the SL is required for obtaining a complete characterization of the complex. (**B**) The ^1^H-*Γ*_2_-CCLS pulse sequence for PRE *Γ*_2_ measurements. The narrow and wide bars represent 90° and 180° hard pulses, respectively. The three ^13^C 180° shaped pulses are 256 μs long Q3 pulse, the first two and the last one shaped pulses are applied to ^13^C′ and ^13^C_α_, respectively. The ^13^C′ 180° shaped pulse may be at either position a or b. When it is at position a, the ^1^*J*_NC′_ is decoupled and reference spectra are acquired. When it is at position b, the ^1^*J*_NC′_ is present and ^13^C′-suppressed spectra are acquired. The flipping angles and phases of the pulses in 3919 are 20.8°_x_, 62.2°_x_, 131.6°_x_, 131.6°_−x_, 62.2°_−x_, and 20.8°_−x_, respectively, and the interval between pulses is 188 μs (=1/d, d is the distance in Hz between center and next null). T = 16.5 ms, Δ = 2.6 ms, G1 = (1 ms, 25.0 G/cm), G2 = (0.3 ms, 5.0 G/cm), G3 = (0.3 ms, 8.0 G/cm), G4 = (1 ms, 15.0 G/cm), and G5 = (1 ms, 10.0 G/cm). The phase cycling scheme is φ1 = (x, −x), φ2 = (x, x, −x, −x), φ3 = 4(x), 4(−x), φ_rec_ = (x, −x, x, −x, −x, x, −x, x). The quadrature detections in *t*_1_ dimension are acquired via States-TPPI of φ1. Constant time mode is used to measure *Γ*_2_, which is $\Gamma_{2}=\mathrm{Ln}\left(S_{1}/S_{2}\right)/\left(\tau_{2}-\tau_{1}\right)$, where $S_{1}$ and $S_{2}$ are signal intensities of a peak measured with $\tau =\tau_{1}$ and $\tau =\tau_{2}$, respectively. 2 × 2 spectra are acquired in an interleave mode via changing relaxation delay *τ* (minimum 2 ms) and changing the ^13^C′ 180° shaped pulse from position a to b, respectively. (**C**) Intra-molecular and inter-molecular PRE measurements of ^15^N-Ubi^K48C^ obtained with the ^1^H-*Γ*_2_-CCLS experiment. The ^1^H_N_-*Γ*_2_ rate plot calculated for K48C mutant conjugate with MTSL in presence of Ubi^WT^ (upper panel C). The ^1^H_N_-*Γ*_2_ rate plot calculated for WT ubiquitin in the presence of Ubi^K48C^-MTSL (lower panel C). (All figures were cited with permission of Springer Nature).

Paramagnetic relaxation enhancements (PRE) have been used extensively to obtain long-distance restraints for structure calculation and to study PPIs for both stable and transient complexes [67,68,69,70,71,72]. In the standard PRE experiment that involves two interacting proteins, the intra-molecular or inter-molecular effects of a paramagnetic center are detected for only one of the binding partners in each independent NMR experiment (Figure 4Ai). To accurately probe these interactions, a minimum of four samples with differing spin label positions as well as reversed labeling schemes are required. Recently, we incorporated the CCLS pulse sequence in the traditional ^1^H_N_-*Γ*_2_ (^1^H_N_-*Γ*_2_-CCLS) [69] that, together with an asymmetric labeling scheme, enables the detection of both intra-molecular and inter-molecular paramagnetic relaxation enhancements (PREs) simultaneously using only one sample [10] (Figure 4B). In this newly proposed strategy, one of the two binding partners must be U-^15^N labeled and the second U-^15^N, ^13^C labeled (Figure 4Aii). We also tested the proposed pulse sequence on the non-covalent, transient dimerization of ubiquitin. Specifically, we studied the complex formed between U-^15^N, ^13^C wild-type ubiquitin and the U-^15^N-spin labeled the K48C mutant. We were able to discriminate intra-molecular and inter-molecular interactions detecting the structural and dynamics changes intrinsic to ubiquitin upon dimerization (Figure 4C). The *Γ*_2_ rates obtained with the new pulse sequence were confirmed to be identical among standard experiments. This work demonstrates that the *Γ*_2_-CCLS PRE experiment is suitable for identifying structural changes occurring in both binding partners upon formation of transient and permanent interactions using a reduced number of samples.

### 2.5. Improving Sensitivity with the G5 Pulse

**Figure 5:**
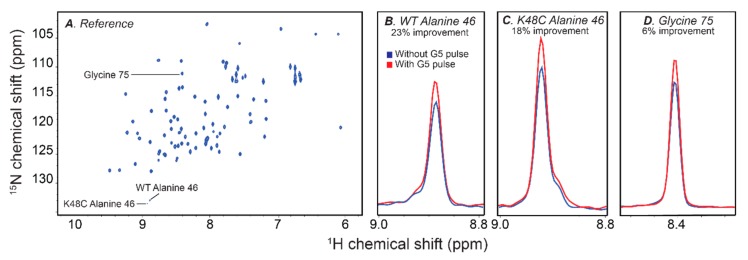
CCLS G5 pulse implementation. (**A**) CCLS ^1^H–^15^N HSQC reference spectrum of ^15^N, ^13^C Ubi^WT^, and U-^15^N Ubi^K48C^ mutant. (**B**) Overlay spectra of the Ubi^WT^ alanine 46 peak demonstrating a 23% signal intensity improvement with the G5 pulse. (**C**) Overlay spectra of the Ubi^K48C^ alanine 46 peak demonstrating an 18% signal intensity improvement with the G5 pulse. (**D**) Overlay spectra of the glycine 75 peak demonstrating a 6% improvement with the G5 pulse. (All figures were cited with permission of Springer Nature).

Advances in NMR methodology (TROSY, deuteration, selective labeling) have allowed for studies of protein-protein complexes approaching 1 MDa [73,74]. However, these studies lack the ability to distinguish one species from another without the preparation of multiple samples. A recent technological advance that can improve nearly any pulse sequence is the universal triply compensated π pulses for high field spectrometers [75,76], which we have incorporated into the CCLS pulse sequence (Figure 5). All inversion and refocusing pulses in the ^1^H and ^15^N channel were replaced with G5 pulses except the ^15^N refocusing pulse in the middle of 3-9-19 water suppression. We were able to improve the signal intensity from 6% to 23% compared to the regular CCLS version. These experiments were performed on the Bruker 900 MHz AVIII spectrometer at 298 K and this enhancement will only be more significant in GHz spectrometers. 

## 3. Conclusions and Perspectives

In this paper, we demonstrate that the CCLS/DCLS pulse sequences enable the study of PPIs through simultaneous inter-leaved detection of all components in a single sample. As we have illustrated, the CCLS and DCLS pulse sequence blocks can be applied to a multitude of well-established experiments (RDC and PRE). Extrapolating from this integration into existing NMR experiments, could NOESY be the next step? The possibility of observing multiple species in a single sample for NOESY experimentation is viable since Anglister et al. [50] has reviewed different spectroscopy and its application to 2D NOESY experiments. However, the pulse sequences are, therefore, limiting sensitivity, which the CCLS/DCLS pulse blocks show promise toward combating. Therefore, reflecting upon the versatility of the CCLS/DCLS pulse block and the associated advantages afforded, we envisage the insertion into other existing NMR experiments to study a wide range of multicomponent systems.
